# Pediatric cylindrical battery ingestion

**DOI:** 10.1055/a-2526-0108

**Published:** 2025-02-26

**Authors:** Maria Boccia, Manuela Pugliese, Marika Cantelli, Alessandro Fierro, Rossella Turco, Piergiorgio Gragnaniello, Alessia Salatto, Ludovica Carangelo, Mariano Caldore, Paolo Quitadamo

**Affiliations:** 1 165474 Department of Translational Medical Sciences, Section of Pediatrics, Federico II University Hospital, Napoli, Italy; 2 9254 Pediatric Gastroenterology and Hepatology Unit, Santobono-Pausilipon Children’s Hospital, Naples, Italy, Santobono Pausilipon Azienda Ospedaliera Pediatrica, Naples, Italy; 3 165474 Clinical Pharmacology and Toxicology Unit, Department of Neuroscience and Reproductive and Odontostomatological Sciences, Federico II University Hospital, Napoli, Italy; 4 9254 Clinical and Translational Research Unit, Santobono Pausilipon Azienda Ospedaliera Pediatrica, Napoli, Italy

**Keywords:** Endoscopy Upper GI Tract, Foreign-bodies, Endoscopy Lower GI Tract, Foreign bodies, Pediatric endoscopy

## Abstract

**Background and study aims:**

Accidental ingestion of batteries is well documented in pediatric medical literature, but very few data exist in pediatric medical literature about ingestions of cylindrical batteries (CBs). The aim of our study was to evaluate the features, clinical presentation and clinical outcome of children who have ingested CBs.

**Patients and methods:**

All children admitted for CB ingestion were retrospectively recruited. Clinical data until hospital discharge were accurately recorded, including child age and sex, ingestion modality, signs and symptoms following ingestion, type of CB, results of neck-chest-abdominal x-ray performed to assess the retention site of CB, outcome of endoscopic removal, and whether performed.

**Results:**

Forty-five children (males/females: 26/19; age range: 7–168 months; mean age ± standard deviation: 42 ± 33.9 months) were enrolled. Of them, 15 of 45 (33.3%) had ingested AA batteries whereas 30 of 45 (66.6%) had ingested AAA batteries. CBs were retained in the esophagus in two of 45 children (4.4%), in the stomach in 19 of 45 children (42.2%), and in the duodenum or beyond in the remaining 24 of 45 children (53.3%). None of the patients who underwent endoscopic removal (12/45) had any esophageal or gastric mucosal lesions. No cases of intestinal perforation or surgical complications were reported.

**Conclusions:**

According to our study data, conservative management may be advised for the majority of cases of CB ingestion. However, we acknowledge that CB should be timely removed whenever they are A23 or A27 type, damaged prior to ingestion, in cases of multiple ingestion, whenever retained in the stomach for a prolonged period, or whenever a child complains about any clinical signs or symptoms or had undergone prior abdominal surgery.

## Introduction

Foreign body (FB) ingestion is a challenging clinical scenario in pediatric emergency rooms. Unlike adults, almost all FB ingestions in children are accidental and pertain to objects found at home or on playgrounds 1 2 3 . Clinical features might include symptoms such as drooling, dysphagia, fussiness, chest/abdominal pain, feeding refusal, stridor, wheezing, and respiratory distress 4 . More commonly children may be completely asymptomatic and taken for medical care after ingestion is witnessed or suspected by caregivers. The majority of ingested FBs pass uneventfully along the gastrointestinal tract and do not require any intervention. Nevertheless, in some circumstances FBs may cause important morbidity or even mortality, due to gastrointestinal bleeding, ulceration, perforation, mediastinitis, peritonitis, abscess, or fistula formation. Moreover, in case of vomiting, a FB may also be inhaled, causing airway obstruction 5 .

Prompt diagnosis and proper management are crucial to minimize any negative outcomes of FB ingestion. Once ingestion is confirmed, the physician must decide whether intervention is necessary and what degree of urgency is called for. Indications for and timing of endoscopic removal rely on assessment of the size and type of the ingested FB, the gastrointestinal retention site, and presence of clinical symptoms 6 7 .

Among the different FBs, accidental ingestion of batteries is well documented in pediatric medical literature. However, the vast majority of scientific data concerning battery ingestion deal with disk/button batteries, which account for a serious health hazard with life-threatening possible complications 8 9 . Conversely, very few data exist on ingestion of cylindrical batteries (CBs), which are less common (likely due to their size), with fewer reported cases and limited data on clinical outcomes. Indeed, no formal recommendations about management of children who have ingested CBs have been developed because no scientific evidence is available 10 . To date there is a lack of data regarding the rate of complications secondary to CB ingestion and pediatric scientific literature is limited to a few case reports and case series, which report variable complication rates.

Therefore, the primary aim of our study was to retrospectively assess the clinical outcome of children having ingested CBs. Secondary aims were to evaluate the features and clinical presentation of these children.

## Patients and methods

The study was a retrospective analysis of all children aged 0 to 14 years admitted for CB ingestion from January 2013 to December 2022 at the Santobono-Pausilipon Children’s Hospital in Naples, third-level referral endoscopic center in Campania region, Italy. The only inclusion criterion was recent ingestion of any type of CB. No exclusion criteria were considered, except for age range.

To investigate features and outcomes of children who had ingested CBs, medical records ware reviewed for all enrolled patients. Of note, demographic and clinical data included child age, sex, ingestion modalities, possible signs and symptoms following ingestion, and information about type of CB ingested (AA or AAA). Moreover, we collected results of diagnostic imaging tests performed to detect the FB retention site and outcome of gastroscopy and whether it was performed for FB removal. Finally, length of hospital stay, time until CB passing in the stools, along with possible short- and long-term clinical complications were recorded for each patient, with special reference to possible prolonged retention and wall perforation during intestinal passage.

Study data were entered into Excel spreadsheets (Microsoft Inc., Washington, United States) and analyzed with GraphPad PRISM software 5.1 (GraphPad Software Inc., California, United States) and R 3.6.0 software environment for statistical computing. Quantitative variables were expressed as mean ± standard deviation whereas frequencies and percentages were used for categorical variables.

The study was approved by the “Cardarelli-Santobono” independent Ethics Committee and was conducted in accordance with Declaration of Helsinki and Guidelines for Good Clinical Practice. In full compliance with current privacy regulations, personal patient demographic data were not recorded.

## Results

Over the study period, 5254 children were admitted for FB ingestion, of whom 45 (0.9%) had ingested a CB ( Table 1 ). Of them 26 were male (57.8%) and 19 were female (42.2%). The age range of children at the time of ingestion was 7 to 168 months, with a mean age of 50 months, a standard deviation of 33.9 months, and a median age of 42 months. The majority of children were toddlers (1–3 years old) (19/45, 42.2%), whereas 15 of 45 (33.3%) were school-age children (5–12 years old) and nine of 45 (20%) were preschool-age children (3–5 years old), one of 45 (2.2%) was an infant (< 1 year old), and one of 45 (2.2%) was adolescent (12–18 years old).

**Table:** **Table 1** Main baseline features of enrolled children.

Boys, n (%)	26/45 (57.8)
Female, n (%)	19/45 (42.2)
**Age group**	
Age in months, mean ± SD (range)	50±33.9 (7–168)
Infants (< 1 year), n (%)	1/45 (2.2)
Toddlers (1–3 years), n (%)	19/45 (42.2)
Pre-school age children (3–5 years), n (%)	9/45 (20)
School-age children (5–12 years), n (%)	15/45 (33.3)
Adolescents (12–18 years), n (%)	1/45 (2.2)
**Symptomatic presentation, n (%)**	6/45 (13.3)
Dysphagia, n (%)	3/45 (6.7)
Food refusal, n (%)	2/45 (4.4)
Marked irritability, n (%)	2/45 (4.4)
Lack of appetite, n (%)	1/45 (2.2)
**Asymptomatic presentation, n (%)**	39/45 (86.7)
**Underlying chronic diseases, n (%)**	7/45 (15.6)
Delayed psychomotor	
development, n (%)	4/45 (8.9)
Asthma, n (%)	2/45 (4.4)
Cow’s milk protein allergy, n (%)	1/45 (2.2)
**Retention site of foreign body**	
Esophagus, n (%)	2/45 (4.4)
Stomach, n (%)	19/45 (42.2)
Duodenum or beyond, n (%)	24/45 (53.3)
**Type of batteries ingested**	
AA, n(%)	15/45 (33.3)
AAA, n (%)	30/45 (66.6)

Chronic medical conditions affecting the enrolled children were delayed psycho-motor development (4 cases), asthma (2 cases) and cow’s milk protein allergy (1 case). One-third of the children (15/45, 33.3%) had ingested AA batteries, whereas two-thirds (30/45, 66.6%) had ingested AAA batteries. All ingestions were reported as accidental and were witnessed by parents or caregivers. Mean time from ingestion to presentation was 105 ± 59 minutes.

On admission, 39 of 45 patients (86.7%) were asymptomatic. The remaining six patients (13.3%) complained of one or more of the following symptoms: dysphagia, food refusal, marked irritability, or lack of appetite.

**Fig. 1:**
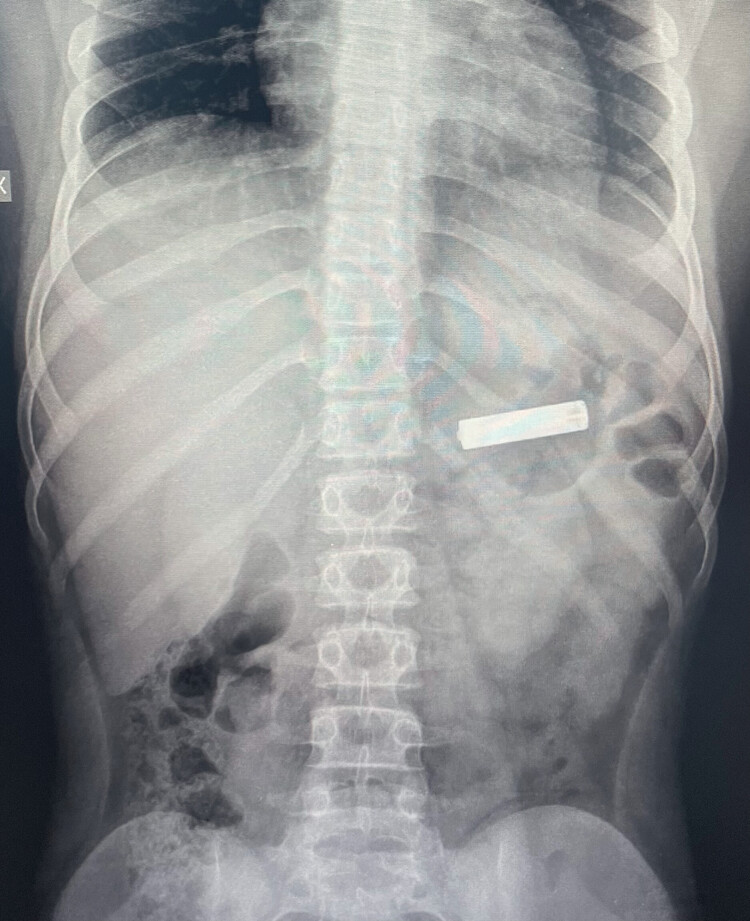
Chest-abdominal x-ray showing a gastric retained cylindrical battery.

**Fig. 2:**
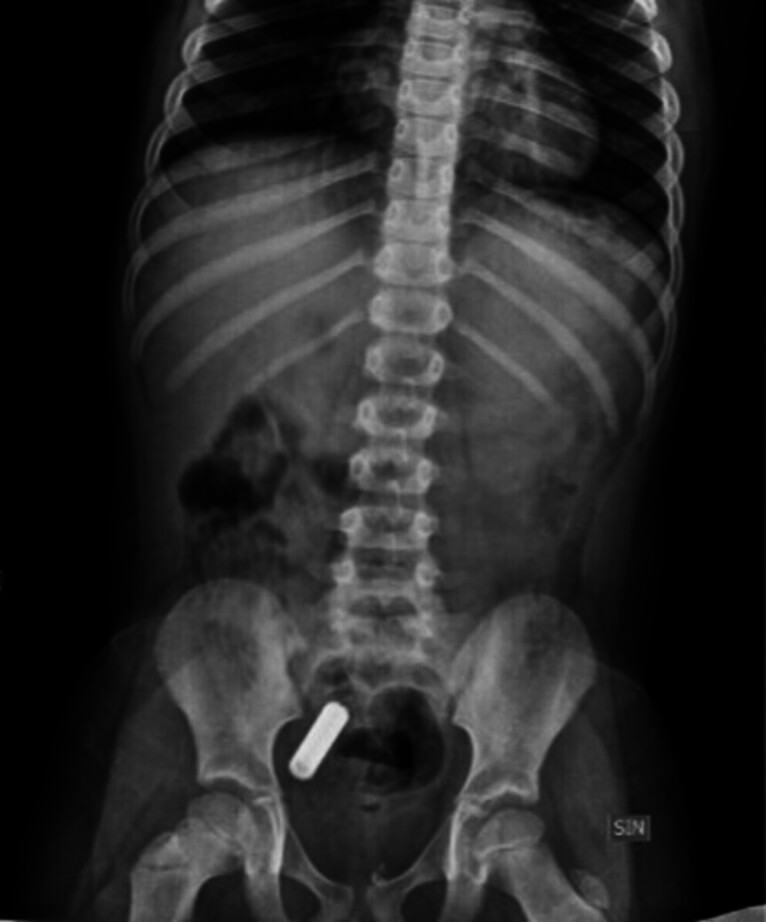
Chest-abdominal x-ray showing an intestinal retained cylindrical battery.

A neck-chest-abdominal x-ray was performed for all patients to assess presence and retention site of the FB. CBs were retained in the esophagus in two of 45 children (4.4%), in the stomach in 19 of 45 children (42.2%), and in the duodenum or beyond in the remaining 24 children (53.3%) ( Fig. 1 and Fig. 2 ).

Children with esophageal CB retention were a girl and a boy aged 64 and 74 months, respectively. They had ingested an AA and an AAA battery and both complained of dysphagia. Children with CBs retained in the stomach were 12 boys and 10 girls with a mean age of 49.8 months. Of them, four of 19 (21%) were symptomatic at admission. Twelve of 19 (63.2%) had ingested AAA batteries and seven of 19 (36.8%) AA batteries. Finally, children with intestinal CB retention were 13 boys and 11 girls with a mean age of 49.3 months. Among them, only two of 24 (8.3%) complained dysphagia and one of 24 (4.2%) refused food. The remaining children were asymptomatic.

Endoscopic removal was performed in 12 patients, two of whom had a CB retained in the esophagus and 10 had a CB retained in the stomach. CB removal was obtained using different devices, including loops and baskets. None of the patients who underwent endoscopic removal had any esophageal or gastric mucosal lesions.

The only patients who were hospitalized were the 12 patients who underwent endoscopic removal. Their average hospitalization stay was 18.3 hours (range 12–24 hours). None of the patients received a prescription for medical treatment and there were no side effects noted.

Children not undergoing CB endoscopic removal were followed up until expulsion. There were no cases of intestinal perforation or surgical complications and no patients underwent CB surgical removal due to lack of intestinal progression. Only spontaneous passage and expulsion were observed. Overall mean ± standard deviation time between CB accidental ingestion and rectal expulsion (whenever not endoscopically removed) was 28.9 ± 8 hours (range: 22–45 hours). Children with chronic constipation were given fecal softeners in order to hasten FB intestinal transit, with no adverse effects reported.

## Discussion

Accidental ingestion of CBs is a current issue in the pediatric age group. According to our study data, CBs are involved in fewer than 1% of all FB ingestion cases in which medical advice is sought. Toddlers accounted for the majority of ingestions, which is likely due to their well-known proclivity for putting objects in their mouths. On the other hand, infants accounted for very few of the cases in our study, due to their inability to move freely and grasp objects from the environment as well as the large size of CBs. It is worth noting that we have not recorded any deliberate ingestions, which are more common in adult and psychiatric populations 11 12 .

Within our study sample, none of the children who had ingested CBs had any significant mucosal lesions or complications related to FB intestinal passage. These data tend to confirm that CBs likely act as blunt FBs. As such, they have to be promptly endoscopically removed in case of esophageal retention or whenever patient age or a previous surgery on the gastrointestinal tract increases the likelihood of difficult intestinal transit. Indeed, timely radiological evaluation is mandatory to detect the site of retention and guide patient clinical management. On the other hand, children with CBs retained in the stomach or beyond it can be followed up until expulsion, except when there are concerns about intestinal progression.

To date, existing international guidelines on management of children who have ingested FBs only briefly cover the possibility of CB ingestion because only limited scientific data are available. The decision about whether to remove a CB from the gastrointestinal tract is left to the individual subjective evaluation of the healthcare professional, depending on several factors, including battery size, retention site, and whether the child is experiencing any symptoms.

Of note, the European Society of Gastrointestinal Endoscopy (ESGE) and European Society for Paediatric Gastroenterology Hepatology and Nutrition (ESPGHAN) 2017 guidelines recommend urgent endoscopic removal for single CB ingestion when impacted in the esophagus and as soon as possible elsewhere in the gastrointestinal tract when the child is symptomatic. ESGE/ESPGHAN suggests that a single CB in the stomach can be observed and the child monitored as an outpatient and followed by x-ray for 7 to 14 days after ingestion if the battery is not passed in stool. Once the batteries pass the pylorus, they almost universally pass the remainder of the gastrointestinal tract without incident 13 .

The American Society for Gastrointestinal Endoscopy (ASGE) guidelines suggest that CBs in the stomach without signs of gastrointestinal injury may be observed for as long as 48 hours. CBs that remain in the stomach longer than 48 hours should be removed 14 .

Our literature review identified only a few papers reporting outcomes of children who had ingested CBs. In 1985, Litovitz analyzed 125 battery ingestions, including 119 button batteries and six cylindrical cells. They reported that all the CBs passed through the gastrointestinal tract spontaneously, without endoscopic or surgical intervention 15 . Moreover, in 2010, Litovitz et al reviewed 8.648 battery ingestion cases, 487 of which were CB ingestions 16 . Data were analyzed from the source National Battery Ingestion Hotline (NBIH) from July 1990 to September 2008. Very few clinical data were available, yet no fatal cases or major injuries were reported among the latter group. More recently, in 2021, Akilov et al described 23 children who ingested CBs enrolled during the years 2014 to 2019. CBs were retained in the esophagus in no patients, in the stomach in 11 patients, and beyond the stomach in the remaining 12 patients. Of the 23 children who ingested CBs, 11 batteries were endoscopically removed and in 55%, mild gastric mucosal injuries were found 17 .

Of note, five case reports with different clinical outcomes have recently been published. In 2014, a 12-month-old girl who had accidentally ingested a CB was diagnosed with two ulcers, approximately 10 to 15 mm in diameter, on the front and back walls of the stomach together with several small erosions on the greater curvature. Yet the ingested battery was identified as a type A23, which consists of eight button alkaline cells (based on a manganese dioxide chemical system) bound together to form a cylinder that is 10 mm in diameter and 28 mm long 18 . In 2017, a 17-year-old girl was reported to have ingested three CBs. Endoscopic removal was performed 14 hours after ingestion. Both ends of the batteries had eroded and there was evidence of significant gastric ulceration and gastritis in the stomach due to caustic acid damage 19 . Finally, in 2023, a 12-year-old boy was reported to have eroded intestinal mucosa, which caused the small bowel wall to be thinned out after CB ingestion. Enterotomy was performed over the thinned-out small bowel wall, exposing the negative terminal of the CB, which had very likely been ingested already consumed and eroded 20 .

In 2021, a case of an 11-month-old female who ingested the internal alkaline contents of an AA battery was reported. In an alkaline AA battery, the internal contents are a mixture of zinc-manganese dioxide and sodium or potassium hydroxide. Approximately 14 hours after ingestion, esophagogastroduodenoscopy showed extensive ulceration and adherent fibrin in the lower esophagus, with an area above the lower esophageal sphincter with a dark appearance indicative of recent bleeding or necrotic tissue 21 .

A similar case was described in 1996. A 4-year-old boy sustained an esophageal burn due to ingestion of an alkaline substance from a leaking cylindric 3.3-cm-diameter battery, by putting the battery to his mouth and sucking the caustic solution 22 .

According to our study data, conservative management may be advised for the majority of CBs cases because CBs do not easily get stuck in the esophagus and move smoothly through the gastrointestinal tract, causing minimal mucosal injury due to their shape and structure. Indeed, the positive/negative terminals are separated by distance so tissue connecting both poles is hard to accomplish, and even if it did, the amount of resistance cannot be overcome to allow current to pass. Moreover, CBs have only 1.5 volts compared with 3-volt lithium coin/button batteries.

In our opinion, ingestion of eroded and consumed CBs should dictate different management because corrosive and toxic damage has been reported. Such damage can occur if battery casing integrity is compromised at time of ingestion, as well as from continuous acid attack from gastrointestinal contents over weeks rather than days, as a result of battery leakage. In these cases, ingestion of CBs may result in significant consequences such as bowel perforation and intestinal obstruction. Moreover, particular attention should be paid to children who have ingested multiple CBs and those with prolonged CB gastric retention, because they are more likely to be predisposed to gastric injury.

Finally, clinicians should be aware of the existence of particular kinds of CBs named A23 and A27, which consist of eight individual alkaline button cells enclosed in a wrapper. Ingestion of both A23 and A27 batteries should be managed as multiple disk battery ingestion, as they actually are.

To the best of our knowledge, this is the first study specifically focused on CB ingestion in a pediatric population. We acknowledge only a few limitations, related to the study’s single-center nature and the limited study sample, although ours is one of the largest cohorts ever published due to the rare occurrence of CB ingestion.

Given the rarity of CB ingestion, lack of clinical data, and variety of CBs commercially available, more research is needed to develop specific recommendations for management of their ingestion.

## Conclusions

Unlike ingestion of disk batteries, ingestion of CB is an uncommon medical presentation with a paucity of published data on clinical outcomes. Our study data lead us to argue that CB ingestion can be managed conservatively because they easily pass through the gastrointestinal tract, given their shape, and pose a low threat of caustic damage, owing to their structure. However, clinicians should be aware of particular conditions for which this recommendation should not apply. Of note, CB should be timely removed whenever they are A23 or A27 type, whenever they were damaged prior to ingestion, in case of multiple ingestion, whenever they are retained in the stomach period, and whenever the child complains about any clinical signs or symptoms or has a history of prior abdominal surgery.
